# How Long Do We Have? A Retrospective Review of Palliative Extubation in the Burn Unit

**DOI:** 10.1093/jbcr/iraf149

**Published:** 2025-07-25

**Authors:** Hannah M Jones, Colette Galet, Alexander Kurjatko

**Affiliations:** Department of Surgery, Division of Acute Care Surgery, University of Iowa, Iowa City, IA 52242, United States; Department of Surgery, Division of Acute Care Surgery, University of Iowa, Iowa City, IA 52242, United States; Department of Surgery, Division of Acute Care Surgery, University of Iowa, Iowa City, IA 52242, United States

**Keywords:** extubation, palliative, burn, death, predictors

## Abstract

Palliative extubation is the termination of mechanical ventilation to allow for a natural death when a patient’s goals no longer align with maintenance of ventilator support. Anticipating a patient’s survival time after palliative extubation is important when counseling patient families and can facilitate individualized palliative care and organ donation processes. This has not been explored in burns. Herein, we aimed at identifying factors associated with death within 1 h of palliative extubation within our adult burn unit population. This is a retrospective case–control study. Adult patients who underwent palliative extubation from July 10, 2015 to June 30, 2023 were included. Demographics, comorbidities, injuries, and clinical parameters were collected. Variables with a *P*-value ≤.1 in univariate analysis as well as age, sex, and TBSA burned (%) were included in the multivariate analysis to identify factors associated with death within 1 h of palliative extubation. *P* < .05 was considered significant. Forty-seven patients underwent palliative extubation; 25 (53.2%) died within 1 h. On univariate analysis, a higher number of vasoactive medications, Sequential Organ Failure Assessment (SOFA) scores, anion gap, phosphorus, lactic acid levels, lower mean arterial pressure, acidosis, and the absence of a history of cerebrovascular disease were associated with death less than 1 h of palliative extubation. Multivariable analysis demonstrated that relatively higher SOFA scores (OR = 2.851 [1.173-6.931]) and anion gaps (OR = 1.687 [1.014-2.806]) were associated with death within 1 h of palliative extubation. While some uncertainty will always be present when predicting time to death after palliative extubation, our study provides a guide to be used in goals of care discussions.

## INTRODUCTION

Burns are life-threatening injuries that may result in death from burn shock, sepsis, acute respiratory distress syndrome, organ failure, anoxic brain injury, or other morbid conditions.^1–4^ When these conditions become overwhelming, patients, families, and practitioners in the intensive care unit (ICU) may be faced with the challenging choice of terminating life-sustaining measures.^5^ Palliative extubation is the termination of mechanical ventilation to allow for a natural death when a patient’s goals no longer align with maintenance of ventilator support.^6^ This process is complex, necessitating effective goals of care conversations and the coordination of interdisciplinary teams and patient families.^7^

The negative psychological impact of the death of a family member in the ICU has been studied by psychologists and intensivists.^8^ Studies have correlated the death of a loved one in the ICU and the degree of involvement in decision-making during the process with depression and posttraumatic stress disorder of family members.^9^ Additionally, family members who reported poor communication with ICU providers have been shown to have an increased incidence of complex grief following the death of a family member in the ICU.^8^ Effective communication from ICU providers and accurate prognostications has been shown to enable families to make informed decisions, cope with uncertainty, and ease distress.^10^ Evidence-based measures for estimating time to death (TTD) after palliative extubation can improve prognostication and equip providers with guidance during goals of care conversations. While scoring systems such as the revised Baux (rBaux) score have demonstrated value in predicting overall mortality among those suffering burns, there is no model that currently exists to anticipate how long a patient will live following palliative extubation.^11^

Predicting the TTD following palliative extubation also has importance among patients who are eligible for organ donation. Organ donation after circulatory death is a growing source for transplantation.^12^ One of the most important components of donation after circulatory death is the reduction of ischemia time after withdrawal of life-sustaining measures.^13^ Several studies have therefore attempted to model risk factors that predict TTD within 1 h of palliative extubation for the purpose of optimizing organ donation.^13–15^ This retrospective study was designed to identify predictors for TTD after palliative extubation in burn patients.

## METHODS

### Ethical statement

Our Institutional Review Board approved this retrospective study (IRB # 201712728). A waiver of consent was approved for all subjects.

### Institutional practice

The burn faculty has a discussion with patients and their families focused on patterns of injury, projected treatment plans, and anticipated complications upon admission to the burn unit. Further formal discussions are held with patients and their families weekly for those who are critically ill, have a burn size greater than 20% TBSA, or develop significant clinical changes. Palliative care services and the regional organ donor network are consulted at the discretion of the burn surgery faculty based on projected clinical outcomes. When palliative care participates in patient care, its service meets with patients and their families daily to ensure their needs are being met. Palliative care may be present during the palliative extubation of a patient to provide support, depending on their availability. If a patient lives beyond an hour following palliative extubation, they may be relocated to a palliative care ward where comfort goals for end-of-life can be tailored to. For patients whose surrogate decision-makers elect for organ donation following terminal extubation, successful organ procurement requires death within 1 h after extubation. As such, we selected a TTD of 60 min as the primary endpoint of the study.

### Study design

Our institution’s burn registry was queried retrospectively to identify all burn patients aged 18 and older admitted from July 1, 2015 to June 30, 2023 who had been intubated for any period of time beyond the operating room. Ventilated patients who were extubated as part of end-of-life care were included in our study. This study follows the Strengthening the Reporting of Observational Studies in Epidemiology reporting guidelines for cohort studies.^16^^,^^17^

### Data collection

Demographics, comorbidities, burn injury information, hospital course, vitals, respiratory parameters, and laboratory variables collected are presented in Table 1. Vital signs and laboratory values measured were at the time closest to the palliative extubation. Need for ECMO, CRRT, and CPR was collected if the patient needed any of these during hospitalization. The rBaux score was calculated based on the clinical information collected.

**Table 1:** Variables Collected

**Category**	**Variable**	**Abbreviation**
Demographics	Age	
	Sex	
	Race	
	Body Mass Index	BMI
Comorbidities	Smoker status	
	Chronic obstructive pulmonary disease	COPD
	Chronic heart failure	CHF
	Diabetes Mellitus	DM
	Myocardial infarction	MI
	cerebrovascular disease	CVD
	peripheral vascular disease	PVD
	Dementia	
Burn injury information	Mechanism of injury	MOI
	Presence of inhalation injury	
	Total burn surface area	TBSA
	Second degree burn surface area	
	Third degree burn surface area	
	Burn injury body location	
	Circumferential burn	
Hospital course	Hospital length of stay	LOS
	Ventilator days	
	Prehospital intubation	
	First 24-h fluid totals	
	Escharotomy	
	Canthotomy	
	Surgery performed	
	Tracheostomy	
	Amputation	
	Pressor use	
	Paralytic use	
	Continuous renal replacement therapy	CRRT
	Extracorporeal membrane oxygenation	ECMO
	Blood transfusion	
	Palliative care consulted	
	Date of palliative care consult	
Patient vitals	Sequential Organ Failure Assessment	SOFA
	Temperature	
	Heart rate	
	Respiratory rate	
	Mean arterial pressure	
Respiratory parameters	Oxygen saturation	SPO_2_
	Partial pressure of oxygen	paO_2_
	Partial pressure of carbon dioxide	paCO_2_
	Positive end-expiratory pressure	PEEP
	Fraction of inspired oxygen	FiO_2_
	Tidal volume	
	Minute ventilation	
	PaO_2_/FiO_2_ ratio	
	SPO_2_/FiO_2_ ratio	
Laboratory variables	pH	
	Anion gap	
	Phosphorus	
	Lactate	

This table describes the variables that were collected for this study.

### Statistical analysis

Normality was assessed using the Kolmogorov–Smirnov test for all continuous variables. All non-normally distributed continuous variables are presented as median and interquartile range. Chi-square and Fisher’s exact test were used to compare categorical variables, while Mann–Whitney *U*-test was used for continuous variables. Variables with a *P*-value ≤.1 in univariate analysis as well as age, sex, and TBSA (%) were included in multivariate models. Binary logistic regression was conducted using the forward Wald approach. Goodness of fit was assessed using the Hosmer–Lemeshow test. All analyses were performed using SPSS 28.0 (IBM), and *P* < .05 was considered significant.

## RESULTS

### Patient characteristics

A total of 360 patients were intubated for any period of time outside of the operating room during the study period. Forty-seven patients (13.1%) transitioned to comfort care involving palliative extubation; 25 (53.2%) died within 1 h postpalliative extubation (median time: 16 [9.5-23.5] min. Twenty-two patients (46.8%) died more than 1 h postpalliative extubation. Only 5 patients (10.6%) lived longer than 24 h (median time: 2 [1.5-7] days). Most patients died in the burn unit or surgical ICU, although 4 patients were transferred to the palliative service (median TTD: 2885 min), and one was discharged to a hospice facility (TTD: 5 days).

As shown in Table 2, there were no significant differences in demographics, comorbidities, or burn injury between patients who died within 1 h vs those who died over an hour after palliative extubation.

**Table 2:** Patient Characteristics

	**Death > 60 min**	**Death within 60 min**	
**Variables**	***n* = 22**	***n* = 25**	***P*-value**
Demographics			
Male, *n* (%)	16 (72.7)	18 (72)	>.999
Age, median [IQR]	71 [65-77.5]	66 [55-78]	.326
BMI, median [IQR]	31.1 [25.5-38.2]	26.6 [21.4-36.5]	.227
Comorbidities			
Smoker, *n* (%)	11 (50)	10 (40)	.564
COPD, *n* (%)	9 (40.9)	8 (32)	.558
Diabetes mellitus, *n* (%)	8 (36.4)	5 (20)	.328
Myocardial Infarction, *n* (%)	9 (40.9)	9 (36)	.771
Peripheral vascular disease, *n* (%)	2 (9.1)	0	.214
Chronic heart failure, *n* (%)	4 (18.2)	4 (16)	>.999
Cerebrovascular disease, *n* (%)	3 (13.6)	0	.095
Dementia, *n* (%)	0	0	
Injury information			
% TBSA, median [IQR]	31.5 [17.8-38.2]	46 [17-63.6]	.364
% Second degree burn, median [IQR]	13 [5-25]	7 [2-19]	.535
% Third degree burn, median [IQR]	11.8 [0.8-34]	34.5 [3-51]	.438
Inhalation injury, *n* (%)	15 (68.2)	13 (52)	.373
Modified Baux score, median [IQR]	112.8 [95-138.5]	124 [105.6-141.8]	.364

Abbreviations: COPD, chronic obstructive pulmonary disease; IQR, interquartile range; TBSA, total burn surface area.

### Clinical and laboratory test information

Table 3 summarizes clinical and laboratory information. Patients who died within 1 h postpalliative extubation were prescribed more vasopressor medications than those who died after 1 h, though there was no difference in the proportion of patients on vasopressors between the groups. Their SOFA scores were significantly higher (9 [7.5-11] vs 7.5 [5-8]; *P* = .009) while their mean arterial pressure (56 [36.5-68.3] vs 67.5 [59.5-76.8]; *P* = .039) and pH (7.23 [7.12-7.32] vs 7.37 [7.32-7.43]; *P* = .001) were significantly lower than those of the patients who died more than 1 h postpalliative extubation. Their blood level of anion gap (16 [9.8-20.3] vs 10 [7.0-13.0]; *P* = .022) and lactate (4 [2-8.4] vs 2 [1.6-3.5]; *P* = .024) were also significantly higher than that of patients who died more than 1-h postpalliative extubation. No other differences were observed.

**Table 3:** Clinical and Laboratory Information

	**Death > 60 min**	**Death within 60 min**	
**Variables**	***n* = 22**	***n* = 25**	***P*-value**
On vasopressors, *n* (%)	10 (45.5)	16 (64)	.248
Number of vasopressors, median [IQR]	0 [0-1]	1 [0-3]	**.021**
On paralytic, *n* (%)	1 (4.5)	4 (16)	.352
Required CRRT during hospitalization, *n* (%)	3 (13.6)	8 (32)	.179
Required ECMO during hospitalization, *n* (%)	0	2 (8)	.491
Required CPR during hospitalization, *n* (%)	7 (31.8)	9 (36)	>.999
SOFA score, median [IQR]	7.5 [5-8]	9 [7.5-11]	**.009**
Temperature, median [IQR]	37.2 [36.7-37.9]	36.5 [36.03-38.03]	.857
Heart rate, median [IQR]	93[78.5-119]	98 [67-120]	.768
Respiratory rate, median [IQR]	20.5 [16-25]	21 [16-28]	.889
SPO_2_, median [IQR]	96 [88-99.3]	97 [90.5-98.5]	.654
Mean arterial pressure, median [IQR]	67.5 [59.5-76.8]	56 [36.5-68.3]	**.039**
HR/RR, median [IQR]	4.9 [3.4-6.3]	4.5[2.97-6.46]	.889
pH, median [IQR]	7.37 [7.32-7.43]	7.23 [7.12-7.32]	**.001**
paO_2_, median [IQR]	94 [75-154]	113 [84.8-210.3]	.632
paCO_2_, median [IQR]	41 [33-48]	45.5 [38.3-60.75]	.241
Anion gap	10 [7-13]	16 [9.8-20.3]	**.022**
Glucose	166 [145-193]	156 [163.5-209.8]	.461
Phosphorus	3.1 [2.8-4]	4.5 [3-6.9]	.094
Bilirubin	0.75 [0.43-1.18]	0.75 [0.40-1.27]	>.999
Lactate	2 [1.6-3.5]	4 [2-8.4]	**.024**
PEEP, median [IQR]	8 [8-10]	10 [8-12]	.526
FiO2, median [IQR]	0.4 [0.3-0.61]	0.56 [0.4-0.9]	.292
Tidal volume, median [IQR]	510 [415.5-597]	424 [339-485]	.110
Minute ventilation, median [IQR]	10.5 [8-13]	8.5 [6.95-12]	.668
PaO_2_/FiO_2_ ratio, median [IQR]	250 [203-400]	183 [127-437]	.453
SpO_2_/FiO_2_ ratio, median [IQR]	228.8 [150.8-298.3]	172.5 [110.9-231.7]	.284

Abbreviations: CPR, cardiopulmonary resuscitation; CRRT, continuous renal replacement therapy; ECMO, extracorporeal membrane oxygenation; HR, heart rate; IQR, interquartile range; PEEP, positive end-expiratory pressure; RR, respiratory rate; SOFA, sequential organ failure assessment.

Bold *P* value indicates significant *P* value, *P* < .05.

### Outcomes

As shown in Table 4, there were no significant differences in the number of days on a ventilator, hospital length of stay, or complications between patients who died within 1 h vs those who died over an hour after palliative extubation.

### Variables associated with death within 1 h postpalliative extubation

In multivariate analysis, adjusting for variables with a *P*-value ≤0.1 in univariate analysis as well as age, sex, and TBSA (%), increased SOFA score (OR = 2.85 [1.17-6.93], *P* = .021) and anion gap (OR = 1.69 [1.01-2.81], *P* 0.044) were associated with higher odds of dying within 1 h postpalliative extubation.

## DISCUSSION

While many studies have found that the median TTD fell under 1 h after palliative extubation, some have found patients surviving for several hours.^15^^,^^18–27^ In our study, the median TTD after extubation was 26 min. We evaluated several variables, including vital signs, laboratory value, and illness severity scales, to determine potential associations with early death. Our results show that increased SOFA scores and anion gap are associated with death within 1 h after palliative extubation.

Vital signs are a common metric to trend a patient’s clinical well-being. Hypoxia measured through pulse oximetry has been used as a variable in machine learning models with success.^24^^,^^28^^,^^29^ A retrospective analysis of elderly ICU patients demonstrated that a systolic blood pressure less than 90 mmHg was associated with earlier TTD following withdrawal from support.^23^ Huynh et al. found that FiO_2_ > 70% and the requirement of vasopressors are individually associated with earlier TTD, with these variables serving as markers of a patient’s respiratory and hemodynamic instability.^20^ Wind et al. found that the use of controlled mechanical ventilation was the only variable associated with earlier TTD in their cohort, and Long et al identified higher PEEP as a predictor for earlier TTD.^15^^,^^21^ In our study, no vital signs or any ventilator parameters were associated with a patient’s TTD following extubation. While relatively lower mean arterial pressure and a higher number of vasopressors were significantly associated with death within 1 h of palliative extubation on univariate analysis, no association was found on multivariate analysis.

Laboratory values are useful in alerting physicians to a patient’s clinical decline. Prior studies have associated earlier TTD after palliative extubation through findings of low pH and low serum bicarbonate.^24^^,^^30^ While our study found a relationship between acidosis and death within 1 h of extubation on univariate analysis, there was no relationship seen on multivariate analysis. Instead, we found that a relatively higher anion gap was associated with death within 1 h of palliative extubation. Several studies involving diverse patient populations implicate anion gap as a predictive risk factor for mortality.^31–39^ To our knowledge, this study is the first to find a relationship between anion gap and TTD following cessation of life support.

**Table 4:** Outcomes

	**Death > 60 min**	**Death within 60 min**	
**Variables**	***n* = 22**	***n* = 25**	***P*-value**
Ventilator days, median [IQR]	3 [1-6.3]	2 [1-7.5]	.935
Hospital length of stay (days), median [IQR]	3 [1.8-7.5]	3 [1-11.5]	.935
Complications			
Ventilator-associated pneumonia, *n* (%)	1 (4.5)	2 (8)	>.999
Sepsis, *n* (%)	2 (9)	3 (12)	>.999

Abbreviation: IQR, interquartile.

Many scoring systems exist to guide physicians on the severity of illness that patients present with. The Acute Physiology and Chronic Health Evaluation (APACHE II) score is an illness severity score used to evaluate organ dysfunction.^40^ Higher APACHE II scores have been shown to be correlated with death within 60 min of palliative extubation.^27^ One study found that extremes in another illness severity score, the Charlson Comorbidity Index, were associated with TTD within 24 h of extubation.^23^ Specific to burn surgery, the rBaux score is a useful guide to utilize in palliative care discussions with patients and families. However, the rBaux score may not capture the complexities of a patient’s condition and has not been previously studied in the context of TTD following palliative extubation.^41^ Our institution utilizes the SOFA score to track illness severity in burn-injured and traumatically injured patients. The SOFA score was developed to objectively describe organ dysfunction.^42^ Many prior studies have correlated organ dysfunction with mortality in both burn and general ICU populations.^41^^,^^43–45^ Our study identifies that the SOFA score has an association with death within 1 h of palliative extubation. Interestingly, the rBaux score and its components (age, TBSA, and presence of inhalation injury) were not found to have a significant association with TTD.

Long et al. noted that the presence of diabetes is correlated with a shorter TTD, which they hypothesize could be attributed to cardiac autonomic neuropathy facilitating quicker hemodynamic collapse.^21^ However, another study suggests that acute injury seems to play a larger role than chronic medical conditions in earlier death following extubation.^44^ Indeed, we found that medical comorbidities, including smoking status, COPD, CHF, DM, MI, cerebrovascular disease, PVD, and dementia, were not found to be associated with TTD after palliative extubation. However, acute interventions such as ECMO, CRRT, and CPR were also not associated with TTD after palliative extubation. While acute interventions and comorbidities should remain a point of discussion when counseling families and patients on the overall survivability of a burn injury, they do not appear to have a significant effect on TTD after palliative extubation.

This study comes with limitations. Due to the nature of a retrospective design, one limitation of our study was the inability to standardize the time of collection of laboratory and clinical values. A patient’s or family’s decision to proceed to comfort care may not be entirely random, and therefore, selection bias may exist within the cohort. While the variables used in the analysis were the most recent value collected before extubation, standardization of time between collection and extubation could impact the results. Being placed on ECMO or CRRT was recorded, but we did not account for the length of time spent with either treatment strategy. Similarly, undergoing CPR was recorded but the duration of resuscitation and the dosages of medications were not accounted for in our study. While the number of vasopressors was collected in our study, the duration and dosages of the vasopressors were not. Certain medications that may have impacted outcomes, such as opiate or sedative medications, were not accounted for in our study. The mean length of stay in our population was 3 days, which represents the early stages of a burn patient’s hospitalization. These findings therefore may not generalize to patients who undergo palliative extubation further into their hospital course. Our multivariable analysis included 11 variables, which is high. However, we used the forward Wald approach, which is a systematic approach to identify the most important predictors for a binary outcome. This leads to more accurate predictions and a better understanding of the relationships between variables. Finally, our sample size was relatively small, and this study is not intended to be generalized to all burn patients. Despite these limitations, this study develops a foundation for further research into predictive measures of TTD after palliative extubation of burn patients.

## CONCLUSION

To our knowledge, this is the first retrospective cohort study to examine predictors for TTD after palliative extubation in the burn ICU. Higher SOFA score and elevated anion gap at the time of extubation were associated with post-extubation TTD. The rBaux score, which is a common predictor for mortality in burn patients, was not significantly associated with TTD after extubation. More research should be done to further characterize these predictive measures in burn patients.

## Notes

Author contributions: Hannah M. Jones (Conceptualization [equal], Data curation [lead], Investigation, Methodology, Validation, Visualization, Writing—original draft, Writing—review & editing [equal]), Colette Galet (Conceptualization [equal], Formal analysis [lead], Project administration, Resources [equal], Software, Supervision [lead], Validation, Visualization, Writing—original draft, Writing—review & editing [equal]), and Alexander Kurjatko (Conceptualization [lead], Data curation, Investigation, Methodology [equal], Project administration [lead], Resources [equal], Supervision [lead], Validation, Visualization, Writing—original draft, Writing—review & editing [equal])

Funding: None declared.

## References

[1] Bittner E, Sheridan R. Acute respiratory distress syndrome, mechanical ventilation, and inhalation injury in burn patients. *Surg Clin North Am* 2023;103:439–451. 37149380 10.1016/j.suc.2023.01.006 PMC10028407

[2] Kallinen O, Maisniemi K, Bohling T et al. Multiple organ failure as a cause of death in patients with severe burns. *J Burn Care Res* 2012;33:206–211. 10.1097/BCR.0b013e3182331e73 21979843

[3] Williams FN, Herndon DN, Hawkins HK et al. The leading causes of death after burn injury in a single pediatric burn center. *Crit Care* 2009;13:R183. 10.1186/cc8170 PMC2811947 19919684

[4] Zavlin D, Chegireddy V, Boukovalas S et al. Multi-institutional analysis of independent predictors for burn mortality in the United States. *Burns Trauma* 2018;6:24. 30151396 10.1186/s41038-018-0127-y PMC6103989

[5] Leung DYP, Chan HYL. Palliative and end-of-life care: more work is required. *Int J Environ Res Public Health* 2020;17:7429. 10.3390/ijerph17207429 PMC7599788 33065964

[6] Ortega-Chen C, Van Buren N, Kwack J et al. Palliative extubation: a discussion of practices and considerations. *J Pain Symptom Manag* 2023;66:e219–e231. 10.1016/j.jpainsymman.2023.03.011 37023832

[7] Velez DR, Irons TD, Opimo AB et al. SAFE-GOALS: a protocol for goals of care discussions in the intensive care unit. *Trauma Surg Acute Care Open* 2025;10:e001663. 39845996 10.1136/tsaco-2024-001663 PMC11749792

[8] Kentish-Barnes N, Chaize M, Seegers V et al. Complicated grief after death of a relative in the intensive care unit. *Eur Respir J* 2015;45:1341–1352. 10.1183/09031936.00160014 25614168

[9] Gries CJ, Engelberg RA, Kross EK et al. Predictors of symptoms of posttraumatic stress and depression in family members after patient death in the ICU. *Chest* 2010;137:280–287. 10.1378/chest.09-1291 PMC2816640 19762549

[10] Salins N, Dhyani VS, Mathew M et al. Assessing palliative care practices in intensive care units and interpreting them using the lens of appropriate care concepts. An umbrella review. *Intensive Care Med* 2024;50:1438–1458. 39141091 10.1007/s00134-024-07565-7 PMC11377469

[11] Dokter J, Meijs J, Oen IM et al. External validation of the revised Baux score for the prediction of mortality in patients with acute burn injury. *J Trauma Acute Care Surg* 2014;76:840–845. 24553558 10.1097/TA.0000000000000124

[12] Steinbrook R . Organ donation after cardiac death. *N Engl J Med* 2007;357:209–213. 10.1056/NEJMp078066 17634455

[13] Brieva J, Coleman N, Lacey J et al. Prediction of death in less than 60 minutes after withdrawal of cardiorespiratory support in potential organ donors after circulatory death. *Transplantation* 2014;98:1112–1118. 10.1097/TP.0000000000000186 24918619

[14] Bradley JA, Pettigrew GJ, Watson CJ. Time to death after withdrawal of treatment in donation after circulatory death (DCD) donors. *Curr Opin Organ Transplant* 2013;18:133–139. 10.1097/MOT.0b013e32835ed81b 23425786

[15] Wind J, Snoeijs MG, Brugman CA et al. Prediction of time of death after withdrawal of life-sustaining treatment in potential donors after cardiac death*. *Crit Care Med* 2012;40:766–769. 21983365 10.1097/CCM.0b013e318232e2e7

[16] Vandenbroucke JP, von Elm E, Altman DG et al. Strengthening the Reporting of Observational Studies in Epidemiology (STROBE): explanation and elaboration. *Epidemiology* 2007;18:805–835. 18049195 10.1097/EDE.0b013e3181577511

[17] von Elm E, Altman DG, Egger M et al. The Strengthening the Reporting of Observational Studies in Epidemiology (STROBE) statement: guidelines for reporting observational studies. *Epidemiology* 2007;18:800–804. 18049194 10.1097/EDE.0b013e3181577654

[18] de Groot YJ, Lingsma HF, Bakker J et al. External validation of a prognostic model predicting time of death after withdrawal of life support in neurocritical patients. *Crit Care Med* 2012;40:233–238. 10.1097/CCM.0b013e31822f0633 21926586

[19] Hung YS, Lee SH, Hung CY et al. Clinical characteristics and survival outcomes of terminally ill patients undergoing withdrawal of mechanical ventilation. *J Formos Med Assoc* 2018;117:798–805. 29032021 10.1016/j.jfma.2017.09.014

[20] Huynh TN, Walling AM, Le TX et al. Factors associated with palliative withdrawal of mechanical ventilation and time to death after withdrawal. *J Palliat Med* 2013;16:1368–1374. 10.1089/jpm.2013.0142 PMC3822388 24083651

[21] Long AC, Muni S, Treece PD et al. Time to death after terminal withdrawal of mechanical ventilation: specific respiratory and physiologic parameters may inform physician predictions. *J Palliat Med* 2015;18:1040–1047. 26555010 10.1089/jpm.2015.0115 PMC4677508

[22] Mayer SA, Kossoff SB. Withdrawal of life support in the neurological intensive care unit. *Neurology* 1999;52:1602–1609. 10331685 10.1212/wnl.52.8.1602

[23] Pan CX, Platis D, Maw MM et al. How long does (s)he have? Retrospective analysis of outcomes after palliative extubation in elderly, chronically critically ill patients. *Crit Care Med* 2016;44:1138–1144. 26958748 10.1097/CCM.0000000000001642

[24] Winter MC, Day TE, Ledbetter DR et al. Machine learning to predict cardiac death within 1 hour after terminal extubation. *Pediatr Crit Care Med* 2021;22:161–171. 33156210 10.1097/PCC.0000000000002612

[25] Wisniewski HM, Wegiel J. The role of perivascular and microglial cells in fibrillogenesis of beta-amyloid and PrP protein in Alzheimer’s disease and scrapie. *Res Immunol* 1992;143:642–645. 1455056 10.1016/0923-2494(92)80049-q

[26] Yee AH, Rabinstein AA, Thapa P et al. Factors influencing time to death after withdrawal of life support in neurocritical patients. *Neurology* 2010;74:1380–1385. 20421582 10.1212/WNL.0b013e3181dad5f0

[27] Zheng YC, Huang YM, Chen PY et al. Prediction of survival time after terminal extubation: the balance between critical care unit utilization and hospice medicine in the COVID-19 pandemic era. *Eur J Med Res* 2023;28:21. 36631882 10.1186/s40001-022-00972-w PMC9832251

[28] Achanti A, Szerlip HM. Acid-base disorders in the critically ill patient. *Clin J Am Soc Nephrol* 2023;18:102–112. 35998977 10.2215/CJN.04500422 PMC10101555

[29] Sun X, De Brouwer E, Liu C et al. Deep learning unlocks the true potential of organ donation after circulatory death with accurate prediction of time-to-death. *Sci Rep* 2025;15:13565. 40253393 10.1038/s41598-025-95079-7 PMC12009369

[30] Brieva J, Coleman N, Lacey J et al. Prediction of death in less than 60 minutes following withdrawal of cardiorespiratory support in ICUs. *Crit Care Med* 2013;41:2677–2687. 23939359 10.1097/CCM.0b013e3182987f38

[31] Arai Y, Tanaka H, Shioji S et al. Anion gap predicts early mortality after starting hemodialysis in the elderly. *Clin Exp Nephrol* 2020;24:458–464. 31984460 10.1007/s10157-019-01844-0

[32] Chen J, Dai C, Yang Y et al. The association between anion gap and in-hospital mortality of post-cardiac arrest patients: a retrospective study. *Sci Rep* 2022;12:7405. 35524151 10.1038/s41598-022-11081-3 PMC9076652

[33] Chen X, Yang Q, Gao L et al. Association between serum anion gap and mortality in critically ill patients with COPD in ICU: data from the MIMIC IV database. *Int J Chron Obstruct Pulmon Dis* 2024;19:579–587. 38444550 10.2147/COPD.S433619 PMC10911976

[34] Gao Y, Hong Z, Shen R et al. Association between anion gap and mortality of aortic aneurysm in intensive care unit after open surgery. *BMC Cardiovasc Disord* 2021;21:458. 34556051 10.1186/s12872-021-02263-4 PMC8459533

[35] Ji X, Peng S. The association between serum anion gap and all-cause mortality of unselected adult patients: a retrospective cohort study of >20,000 patients. *J Clin Lab Anal* 2023;37:e24818. 36550640 10.1002/jcla.24818 PMC9833973

[36] Sun X, Lu J, Weng W et al. Association between anion gap and all-cause mortality of critically ill surgical patients: a retrospective cohort study. *BMC Surg* 2023;23:226. 37559030 10.1186/s12893-023-02137-w PMC10413518

[37] Xu H, Xia J, Wang A et al. Serum anion gap is associated with mortality in intensive care unit patients with diastolic heart failure. *Sci Rep* 2023;13:16670. 37794229 10.1038/s41598-023-43928-8 PMC10550980

[38] Zhang XB, Shu WB, Li AB et al. The anion gap and mortality in critically ill patients with hip fractures. *Contrast Media Mol Imaging* 2022;2022:1591507. 35854763 10.1155/2022/1591507 PMC9279042

[39] Zhu Y, He Z, Jin Y et al. Serum anion gap level predicts all-cause mortality in septic patients: a retrospective study based on the MIMIC III database. *J Intensive Care Med* 2023;38:349–357. 10.1177/08850666221123483 36066040

[40] Thakur R, Naga Rohith V, Arora JK. Mean SOFA score in comparison with APACHE II score in predicting mortality in surgical patients with sepsis. *Cureus* 2023;15:e36653. 10.7759/cureus.36653 PMC10128886 37113362

[41] Prasad A, Thode HC Jr, Singer AJ. Predictive value of quick SOFA and revised Baux scores in burn patients. *Burns* 2020;46:347–351. 31859098 10.1016/j.burns.2019.03.006

[42] Moreno R, Rhodes A, Piquilloud L et al. The sequential organ failure assessment (SOFA) score: has the time come for an update? *Crit Care* 2023;27:15. 10.1186/s13054-022-04290-9 PMC9837980 36639780

[43] Calles J, Cohen B, Forme N et al. Variation of the SOFA score and mortality in patients with severe burns: a cohort study. *Burns* 2023;49:34–41. 36202683 10.1016/j.burns.2022.09.004

[44] Chen E, Kosinski N, Kaur R. Time to death after compassionate extubation in medical and neuroscience intensive care units. *Heart Lung* 2025;69:185–191. 39486140 10.1016/j.hrtlng.2024.10.005

[45] Ferreira FL, Bota DP, Bross A et al. Serial evaluation of the SOFA score to predict outcome in critically ill patients. *JAMA* 2001;286:1754–1758. 10.1001/jama.286.14.1754 11594901

